# Sun Compass Orientation Helps Coral Reef Fish Larvae Return to Their Natal Reef

**DOI:** 10.1371/journal.pone.0066039

**Published:** 2013-06-26

**Authors:** Henrik Mouritsen, Jelle Atema, Michael J. Kingsford, Gabriele Gerlach

**Affiliations:** 1 Institut für Biologie und Umweltwissenschaften, Carl-von-Ossietzky Universität Oldenburg, Oldenburg, Germany; 2 Forschungszentrum Neurosensorik, University of Oldenburg, Oldenburg, Germany; 3 Boston University Marine Program, Boston, Massachusetts, United States of America; 4 School of Marine and Tropical Biology and ARC Centre of Excellence for Coral Reef Studies, James Cook University, Townsville, Queensland, Australia; Lund University, Sweden

## Abstract

Reef fish sustain populations on isolated reefs and show genetic diversity between nearby reefs even though larvae of many species are swept away from the natal site during pelagic dispersal. Retention or recruitment to natal reefs requires orientation capabilities that enable larvae to find their way. Although olfactory and acoustically based orientation has been implicated in homing when larvae are in the reef’s vicinity, it is still unclear how they cope with greater distances. Here we show evidence for a sun compass mechanism that can bring the larvae to the vicinity of their natal reef. In a circular arena, pre-settlement larvae and early settlers (<24 hours) of the cardinal fish, *Ostorhinchus doederleini*, showed a strong SSE directional swimming response, which most likely has evolved to compensate for the locally prevailing large scale NNW current drift. When fish were clock-shifted 6 hours, they changed their orientation by ca. 180° as predicted by the tropical sun curve at One Tree Island, i.e. they used a time-compensated sun compass. Furthermore, the fish oriented most consistently at times of the day when the sun azimuth is easy to determine. Microsatellite markers showed that the larvae that had just arrived at One Tree Island genetically belonged to either the local reef population or to Fitzroy Reef located 12 kilometers to the SSE. The use of a sun compass adds a missing long-distance link to the hierarchy of other sensory abilities that can direct larvae to the region of origin, including their natal reef. Predominant local recruitment, in turn, can contribute to genetic isolation and potential speciation.

## Introduction

The persistence of reef fish populations on isolated oceanic islands demonstrates that a significant number of their larvae return to their natal reef after their pelagic dispersal phase [1], [2], [3], [4]. Such “self-recruitment” has been shown using transgenerational otolith tagging [5], [6] and genetic markers e.g. [1], [2]. Failure to recruit to a suitable reef is fatal and the only reef any larva can know initially –by smell, sound, and direction– is its natal reef. This suggests selection for natal homing abilities and there are several arguments that support this idea. Dispersal models better predict the observed recruitment when they include a behavioral component [7], [8], i.e. sustained swimming in an appropriate direction. Swimming capabilities of larval reef fishes are surprisingly strong, particularly late in the pre-settlement period [9], [10]; and sensory guidance based on acoustic [11], [12], [13] and olfactory [14], [15] mechanisms have been suggested. However, both odor halos [14] and acoustic cues [16] become fainter with distance and are unlikely to provide useful directional information beyond a few kilometers. Genetic assignment (e.g. [2], [17]) shows that a significant proportion of larvae end up ten kilometers or more away from their natal reef. Therefore, selection should strongly favor animals that can actively compensate for long distance drift. However, thus far, no plausible mechanism has been demonstrated that could allow pelagic larvae to locate the natal reef from distances beyond a few kilometers after their initial period of passive dispersal during which they are not capable of sustained directional swimming. For longer distance directional movements, animals typically use an innate or acquired compass [18], [19], [20], [21], [22], [23], [24]. Thus, the aim of the present paper is to investigate whether reef fish larvae have a compass mechanism that could be helpful for long-distance homing.

## Results

**Figure 1:**
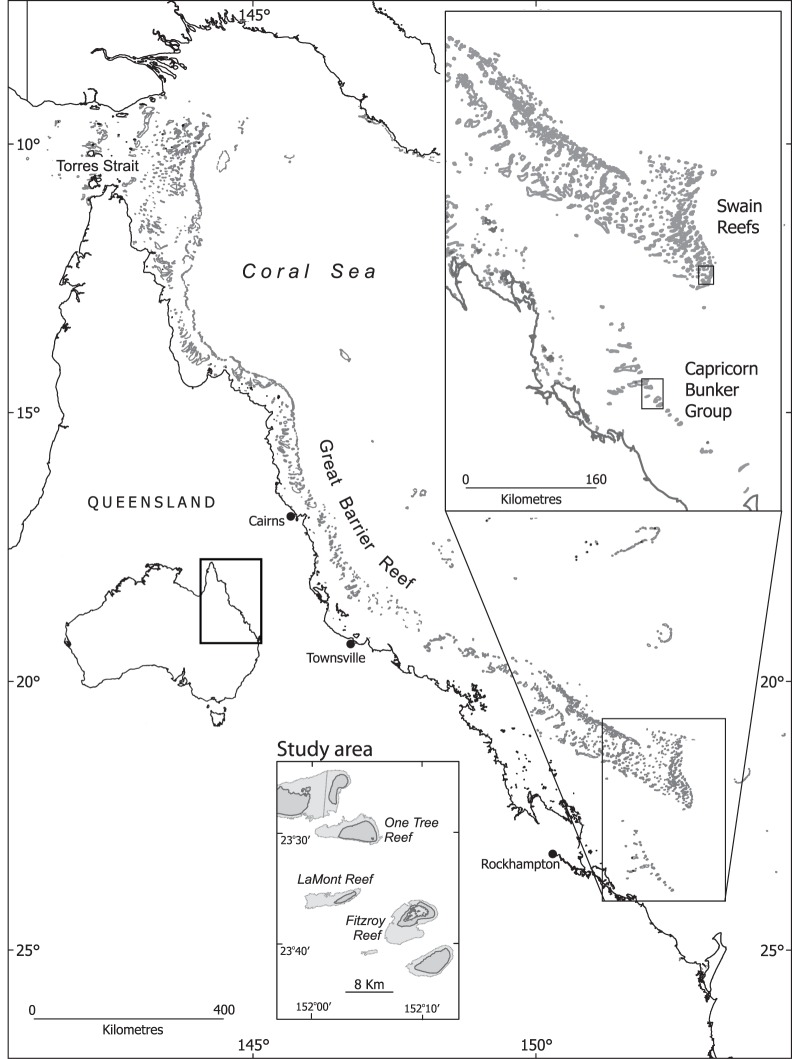
Location of One tree Island. One Tree reef (OTI, 23°30′S, 152°06′E) is one of fourteen reefs in the Capricorn Bunker Group in the southern Great Barrier Reef, Australia. OTI is situated 90 km from the Queensland coast and 5–10 km southeast of neighboring reefs Heron and Sykes.

We tested the directional preferences of just-settling fish (20/Jan and 01/Feb/2011) and pre-settlement fish (20–27/Jan/2012) at One Tree Island in the Capricorn Bunker reef group in the southern Great Barrier Reef, Australia (Figure 1). The directional preferences of each fish were measured by releasing it individually in the centre of a small swimming pool (bowl with diameter 17 cm). After ca. 60 sec, the fish’s geographical position relative to the centre of the bowl was recorded in 30 sec intervals for the next 20 min. Based on the 40 recorded directions, the mean direction of the given fish and test was calculated. To allow for assessment of both intra and inter-individual variance each of 21 individual fish was retested 3–5 times under the natural sunny sky (maximum 75% cloud cover, mostly less than 25%). In total, 88 individual tests were performed under non-clock-shifted conditions (a complete raw data summary of these experiments can be found in the supplementary materials, Table S1–S4 in File S1).

**Figure 2:**
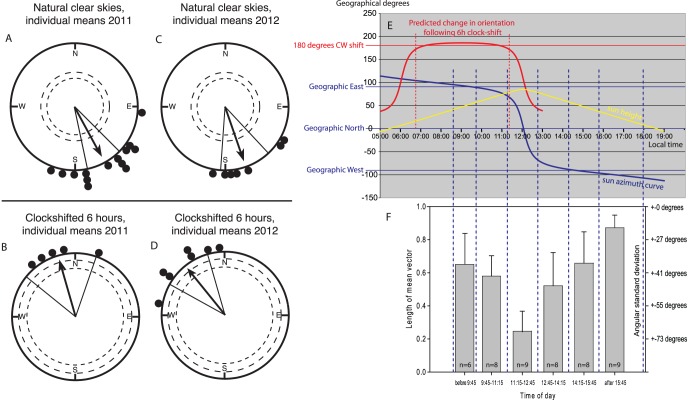
Settling stage *Ostorhinchus doederleini* use a time-compensated sun compass to orient towards SSE. **A**: Fourteen just-settled *O. doederleini* tested under natural sunny skies in 2011 showed a clear orientation towards SSE (mean direction: 152°, r = 0.88, n = 14, p<0.001). **B**: When five of these fish were clock-shifted 6 hours backwards, they turned their orientation by ca. 180° (mean direction: 344°, r = 0.94, n = 5, p<0.01). **C, D**: In January 2012, we repeated the experiments with pre-settlement fish and got very similar results. Seven pre-settlement *O. doederleini* tested under natural sunny skies showed a clear orientation towards SSE (**C**: mean direction: 161°, r = 0.91, n = 7, p<0.001). When all 7 fish were clock-shifted 6 hours backwards, they turned their orientation by ca. 180° (**D**: mean direction: 321°, r = 0.92, n = 7, p<0.001). Each dot at the circle periphery indicates the mean orientation chosen by each individual fish based on the second order average of all tests made with a given fish in the given condition. Arrows indicate the group mean vectors. Inner and outer dashed circles indicate the radius of the group mean vector needed for significance according to the Rayleigh Test (p<0.05 and p<0.01, respectively). Lines flanking the group mean vector indicate the 95% confidence intervals for the group mean direction. **E**: We performed all orientation tests between 20/Jan and 01/Feb. The yellow curve in **E** shows the height of the sun above the horizon at One Tree Island calculated for 25 January 2012 (90° means directly overhead, 0° means that the sun is at the horizon). The blue curve in **E** is the sun azimuth curve at One Tree Island calculated for 25 January 2012. Notice that in the morning until about 11∶15, the sun azimuth is very consistently in the East (117°–77°). Likewise, in the afternoon from 12∶45 onwards, the sun azimuth is very consistently in the West (293°–243°). In contrast, at noon between 11∶15 and 12∶45, the sun is more or less directly overhead (the sun is 78–86 degrees above the horizon, see yellow curve) and the sun azimuth changes by 139 degrees in just 90 minutes. **F** is showing how strongly oriented the individual fish were during tests in the different time intervals. The left y-axis is indicating the length of the mean vector, “r”, calculated by vector addition of the 40 observed directions during a single test of a given individual. The greater the r, the more consistently the fish oriented. The mean vector length is inversely proportional to the angular standard deviation (s = (-ln(r))½) which is indicated on the right y-axis. Figure **F** is aligned exactly under Figure **E** so that the blue dashed lines identify the time range and sun azimuth positions that contributed data to each of the six time blocks. Notice that the fish oriented very poorly during the 11∶15–12∶45 time block, when a sun compass would be very difficult to use because the sun is almost directly overhead and shows an exceptionally rapid change in azimuth (139 degrees in just 90 minutes, i.e. 1.5 degrees/minute). Accurate orientation during this time would require a very precise synchronization of the animals’ internal clock to the specific sun curve. In contrast, late in the afternoon when a sun compass would be particularly easy to use because the sun azimuth changes very slowly and because the sun is close to the horizon, the fish showed extremely directed orientation. The unusual sun curve also means that a 6 hour clock-shift where the animals wake up around midnight and are tested before noon, when they think it is afternoon, leads to an extremely consistent predicted change in orientation of 180 degrees. This is documented by the red curve in Fig. **E**, which shows the predicted clockwise shift in orientation following a 6 hour clock-shift as a function of the time of day during which the fish are tested after being clock-shifted 6 hours. The red curve was calculated as follows: the Sun azimuth at testing time - the sun azimuth 6 hours later. We tested our clock-shifted fish between 06∶45 and 11∶02 (as indicated by the dashed vertical red lines) when the expected orientation of the 6 hrs time shifted larvae predicts a 180 degree shift for this entire 4∶17-hr observation window.

The non-clock-shifted, just-settling fish tested in 2011 spontaneously and highly significantly oriented towards the SSE (Rayleigh Test: mean direction 152°, n = 14, r = 0.88, p<0.001; Figure 2A). This direction is opposite to the persistent NNW drift of the water masses around One Tree Island [2], [25]. An internal replication using pre-settlement fish tested in 2012 showed the same directional preference (Rayleigh Test: mean direction 161°, n = 7, r = 0.91, p<0.001; Figure 2C).

We have direct evidence that long distance NNW dispersal occurs at the testing site. Using DNA microsatellite markers, we could assign the individual larvae, which we had shown to swim SSE in 2011, to adult populations of OTI and to Fitzroy reef located 12 kilometers from OTI in the same SSE direction (Figure 1). None of the larvae were assigned to Heron or Lamont reef populations located to the NW or SW of OTI. Five (31%) larvae were assigned to the OTI population with a mean probability of 78%; the other eight (69%) larvae were assigned to the Fitzroy population with a mean probability of 77% (Table 1). Thus, NNW larval dispersal beyond 10 kilometers (i.e. from Fitzroy) occurs regularly. Therefore, the SSE swimming direction could be an adaptation to counter large-scale dispersal away from the fishes’ natal reef. This begs the question of how they know and maintain this SSE heading.

**Table 1:** Genetic assignment of recently settled larval *O. doederleini* to adult reef populations.

Assigned larva*	Rank 1	Score %	Rank 2	Score %
OTI11,HM1 (cs)	OTI	87.4	F	8.5
OTI11,HM3 (cs)	F	77.7	H	10.5
OTI11,HM4 (cs)	OTI	89.2	F	10.7
OTI11,HM5 (cs)	F	55.9	OTI	41.8
OTI11,HM9	F	92.4	L	7.0
OTI11,HM10 (cs)	OTI	86.3	F	13.1
OTI11,HM11	F	99.6	OTI	0.4
OTI11,HM12	OTI	55.6	F	44.2
OTI11,HM13	F	97.1	OTI	2.7
OTI11,HM19	OTI	80.5	H	14.6
OTI11,HM20	F	64.7	OTI	33.1
OTI11,HM21	F	55.7	OTI	34.1
OTI11,HM22	F	70.6	L	28.4

* One sample (OTI11, HM2) could not be used for genetics. “(cs)” means that this individual was clock-shifted after being tested under non-clock-shifted conditions. Genetic assignment of thirteen post-settlement larvae caught at the One Tree reef (OTI) tested for orientation in the sun compass using five microsatellite markers; the first two ranks (i.e. the two most likely origins of the larvae) and their probability score are shown. Statistical analysis was performed following the Bayesian approach by Rannala [52]. L (Lamont), H (Heron) F (Fitzroy).

During orientation tests at different times of day (the time ranges are indicated by the dashed blue lines in Figure 2E–F and detailed information on each individual test is given in Table S1 in File S1) we noticed that the fish appeared very poorly oriented when tested around noon (Figure 2F). One Tree Island (23°30″ S, 152°05″ E) is part of the Capricorn group of islands, named so because these islands are located on the “Tropic of Capricorn”, where the sun passes directly overhead on 21^st^ of December. In late January on One Tree Island, the sun still passes from very consistent easterly positions before noon, to being almost directly overhead at noon, and to very consistent westerly positions in the afternoon. This leads to a very unusual local sun azimuth curve (see Figure 2E), which is ideally suited to test for sun compass orientation. In the morning from 07∶00 to 11∶00, the sun azimuth is in the East moving only 25 degrees in 4 hours. Likewise, in the afternoon from 13∶00 to 17∶00, the sun azimuth is in the West moving only 28 degrees in 4 hours. In contrast, around noon, between 11∶15 and 12∶45, the sun is more or less directly overhead (78–86 degrees above the horizon) and the sun azimuth changes by 139 degrees in just 90 minutes. This causes problems for the use of a sun compass because the sun azimuth is difficult to determine: the sun is almost directly overhead and the exceptionally rapid change in azimuth would require very precise synchronization of the animals’ internal clock to the local sun curve. The observed lack of orientation around noon suggests that the fish might use a sun compass at other times.

We therefore systematically investigated the consistency of the fish’s directional choices as a function of time of day in 2011 (Figure 2F, a summary table listing the time and orientation result of each individual test contributing to Figure 2F is provided in supplementary materials, Table S2 in File S1). The fish’s directedness, quantified as the length of the mean vector (r) of the 40 recorded headings in each single test, showed a clear dependence on time of day (One-way ANOVA; df = 47, F = 16.1, p<0.001, Figure 2F). In fact, the fish were randomly oriented around noon during the period when a sun compass would be very difficult to determine (11∶15–12∶45, mean r = 0.25; the average length of the mean vector was significantly smaller than in any other time interval, p<0.01 (One-way ANOVA followed by All Pairwise Multiple Comparison Procedures using the Student-Newman-Keuls Method). In contrast, the fish were exceptionally clearly oriented in the late afternoon when the sun is close to the horizon and the sun azimuth is therefore particularly easy to determine (after 15∶45, mean r = 0.87; the average length of the mean vector was significantly larger than in any other time interval, p<0.01). This is also reflected in the negative correlation between directedness of the fish and sun elevation (see Figure S2 in File S1). I.e. the lower the sun, the more directed the individual fish were during each single 20 min test session.

To sum up this section, the striking change in the fish’s directedness as a function of time of day indicates that they use a sun compass (e.g. [22], [26], [27]), since the easier the sun azimuth angle could be determined, the better the fish were able to orient. Sun compass orientation has been considered in fish [28], particularly in mosquitofish [29] and migrating salmon [30], and even though it has been suggested as a potential mechanism [31], [32], there has been no direct experimental demonstration for any reef fish larvae.

Since sun position can only be used as a compass cue if time of day is taken into consideration [22], [26], [27], the key experiment needed to prove whether an animal uses a sun compass to determine its orientation direction, is a clock-shift experiment. One Tree Island is ideally suited for such an experiment. When fish are clock-shifted 6 hour backward (light on at 23∶15, light out at 13∶15) and then tested between 06∶45 and 11∶15 in the morning (see red curve and red dashed lines in Figure 2E), the summer tropical sun azimuth curve means that the sun azimuth is consistently 180±10° different six hours later in the day. Thus, if our fish used a time-compensated sun compass to orient SSE, they should shift their orientation by 180° when clock-shifted 6 hours backward and should thus orient to the NNW.

We therefore clock-shifted five of the 2011 just-settling fish and all the 2012 pre-settlement fish 6 hours backwards (light on at 23∶15, light out at 13∶15). After being clock-shifted for 6 days, we re-tested their orientation under sunny skies before noon (06∶45–11∶02) when the sun was in the East. However, since their shifted inner clock told them it was afternoon, the fish should expect the sun to be towards the West. If the fish use a time-compensated sun compass, they should therefore make a ∼180° orientation “mistake” (red curve in Figure 2E). We conducted 44 individual tests with the 12 clock-shifted fish (for detailed information about each single test, see supplementary Table S3 in File S1).

**Figure 3:**
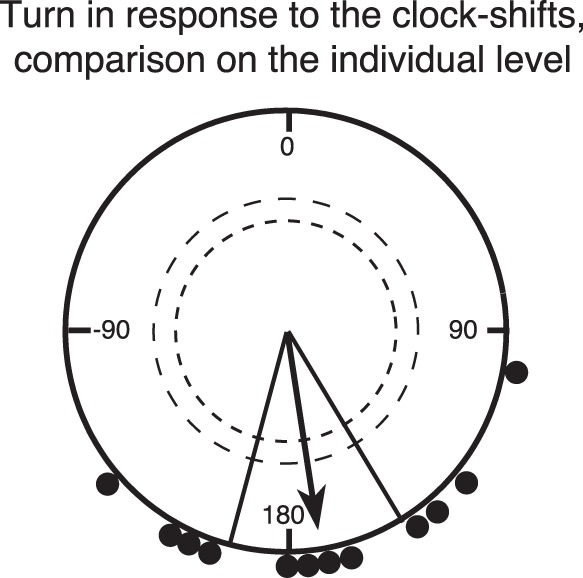
Relative orientation of the individual fish in the clock-shifted condition compared to the same fish’s orientation the non-clock-shifted condition. The fish’s orientation in the non-clock-shifted condition is defined as 0 degrees and the orientation of each individual fish after the clock-shift compared to before the clock-shift is indicated by the dots at the circle periphery (2011 and 2012 fish combined). Thus, a point in 0 degrees would mean no difference between an individual fish’s orientation in the clock-shifted and non-clock-shifted condition. On average, the fish highly significantly shifted their orientation by 172 degrees clockwise in the clock-shifted condition compared to the same fish’s orientation in the non-clock-shifted condition (Rayleigh Test, mean direction = 172, r = 0.83, n = 12, p<0.001. Furthermore, even 99.9% confidence intervals do not include 0 degrees), and the 95% confidence interval (150–195 degrees) amply includes the predicted 180 degrees shift. Raw data in Table S4 in File S1. For description of the circular diagram, see legend to Figure 2.

Indeed, the 6 hours clock-shifted, just-settling fish tested in 2011 oriented significantly towards the NNW (Rayleigh Test: mean direction 344°, n = 5, r = 0.94, p<0.01; Figure 2B), while the nine non-clock-shifted fish continued to orient to the SSE, (Results shown in Figure 2A include data from non-shifted control fish tested at the same post-capture time as the clock-shifted fish, for full details see Table S1 and Table S3 in File S1). The same switch of orientation from SSE to NNW in the clock-shifted condition was observed when we replicated the experiment with pre-settlement fish in 2012 (Rayleigh Test: mean direction 321°, n = 7, r = 0.92, p<0.001; Figure 2D). When the mean directions chosen by each individual fish tested both before and after the clock-shift were compared, it was clear that all individuals strongly shifted their mean orientation in response to the clock-shift (Figure 3). The tests with the clock-shifted fish demonstrate that the fish have an internal clock (“*zeitgeber”*) that they use as part of a time-compensated sun compass to maintain their SSE heading.

## Discussion

**Figure 4:**
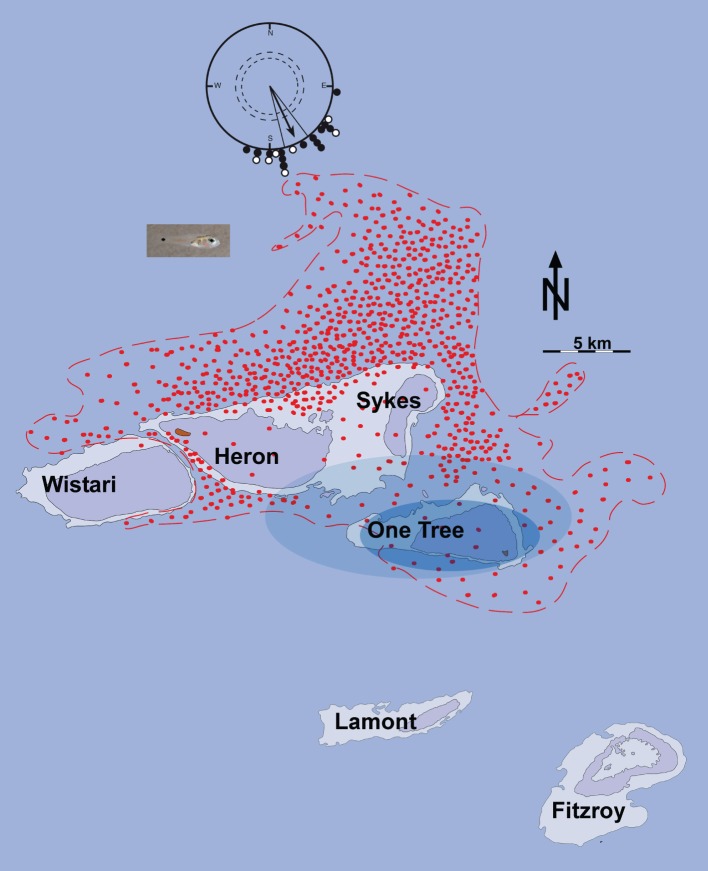
Model illustrating how a time-compensated sun compass could help passively drifted reef fish larvae to relocate their natal OTI reef. Red dots indicate the locations at ebb tide of passively dispersed particles 8 days after release from One Tree Reef according to the dispersal experiments and model calculations (Red dots drawn after Fig. 1C in reference [2]). At flood tide the dots will be displaced 5–7 km WNW. Notice that most larvae considered as passive particles for their first 8 days would be transported significantly to the NNW beyond the odor halo of OTI (idealized odor halos in decreasing intensity blue) and even beyond neighboring reefs to the NNW before they gain sustained swimming capabilities. The polar diagram shows the time-compensated sun compass orientation of just-settled (•) and pre-settlement (o) larvae (Figure 2A and 2C combined: mean direction 155°, n = 21, r = 0.89, p<0.001). One-week old *O. doederleini* larvae (symbolized by the dispersed cloud of red dots) would be more likely to relocate the OTI reef if they used a sun compass to swim actively toward SSE, than if they would swim in random directions. Picture shows settling stage *Ostorhinchus doederleini.*

Although it is not known how far *O. doederleini* larvae disperse from the reef during their pre-settlement phase, genetic data presented here and in [2] indicate that distances beyond 10 kilometers occur regularly. Thus, we consider two likely situations: (1) some larvae remain close to the reef, and (2) others drift further away on prevailing currents and may or may not return to the natal reef (see also Figure 4).

In the first case, OTI larvae would be retained in the reef vicinity within lee side eddies resulting from oscillating tidal currents [25], [33] mostly north of the One Tree reef. For such larvae the sun compass might not be crucial, but it could still be useful to cross the reef crest and settle. In the second case, OTI larvae would be transported away from the tidal eddies by the weak but persistent and unidirectional NNW current resulting from the dominant SE trade winds. The observed SSE directional swimming would be an adaptation to counter long distance NNW drift. It is important to note that the much stronger tidal currents are oscillating East-West around the reef and thus contribute little to long-term, large-scale drift.

For larvae that drift farther away from their natal reef, such as our identified Fitzroy larvae, we suggest the following orientation-relevant chain of events: (I) a passive dispersal phase leading to drift away from the home reef; (II) an actively swimming, sun-compass guided phase to compensate for the drift; and (III) a multi-sensory settlement phase similar to the larvae that were retained near the reef.

We suggest the following pelagic dispersal and settlement scenario. Hatching, *O. doederleini* larvae leave the reef by tidal currents [34]. At the beginning of this passive dispersal phase, the larvae are immersed in natal reef water making imprinting on various home reef cues such as odor and sound possible. A dispersal model based on field-release of neutrally buoyant particles in the currents around the One Tree Reef [2] shows that the centre of the particle distribution on post-release day 8 has drifted to a location of ca. 20 kilometers NW of the One Tree Reef during flood tide and ca. 13 kilometers NNW during ebb tide, i.e. the drift current speed is ca. 13 km/8 days = 1.9 cm/s (Figure 4). Even though large-scale currents will vary with ocean basin phenomena such as ENSO, this drift current is consistent with prevailing trade winds, which are remarkably stable around the study site; South-South Easterly 90% of the time. This predictability would facilitate the emergence of an innate mechanism for sun compass guided directionality by selection, year after year, in favor of the larvae that swim SSE. Since the time-compensation required for a sun compass needs to be learned (because the exact movement of the sun varies greatly with season and latitude), it is likely that this learning takes place during the early dispersal phase.

After about a week of dispersal, the larval swimming capabilities may now be sufficiently effective to start moving in a sun compass-based direction. In young birds, this direction is innate [35], [36], and hatchling sea turtles show adaptive innate reactions to specific magnetic fields [23], [37]). It is likely that the observed SSE swimming direction is innate in OTI *O. doederleini* larvae, but this has not yet been demonstrated.

Could these reef fish larvae realistically compensate for the drift current? Although this species has not been tested for swimming speed, other cardinal fish can maintain speeds of 2 cm/s at hatching and 10 cm/s at settlement [9], which is faster than the slow NNW drift current of ca. 1.9 cm/s observed around OTI [2]. Over a two-week period of perfectly directed sustained swimming the larvae could theoretically cover up to 100 kilometers. However, the larvae are certainly not swimming perfectly SSE and they do not swim all the time. It is nevertheless plausible that sun-compass directed swimming could, in principle, help them return from the 13–20 kilometer drift distance calculated for their early larval period back to the general vicinity of their natal reef. Homing may be facilitated further if the larvae choose favorable currents at different water depths and tidal periods [38], [39]. The constant tidal oscillations generate a permanent odor halo extending several kilometers around each reef, which would further increase the chances of larvae detecting the vicinity of the natal reef. The suggested sun compass guided swimming response is likely to continue until the larvae recognize the odor halo and/or sound of their natal reef, which present a much larger homing target for returning larvae than the reef itself. A larger target allows for greater variance in large-scale currents and homing precision. Surely, the majority of larvae get lost or perish otherwise, but their sun compass directed swimming behavior observed here would increase the likelihood of relocating the area of origin including the natal reef and thus be of evolutionary advantage compared to fish not performing this behavior (Figure 4).

Finally, once the larvae are in the vicinity of their natal reef, we expect them to start using other more local homing cues such as sound [11], [13], [40] and/or odor [14]. The succession of orientation mechanisms suggested here for reef fish larvae is similar to the innate mechanisms used by birds and monarch butterflies during migration: first a compass mechanism based on global cues for the long-distance navigation phase followed by a homing process based on more local cues (e.g. [22], [24], [35], [36]).

In conclusion, here we provide data documenting that reef fish larvae possess and can use a time-compensated sun compass. Our experiments include the critical clock-shifted condition and an internal replication performed in two different years. Our data show that both pre-settlement and recently settled larvae of *O. doederleini* can use a time-compensated sun compass to keep a consistent direction. This direction seems adapted to compensate for drift away from the natal reef and its surroundings. This orientation mechanism, well-known in various terrestrial animals, is well-suited to explain how dispersing fish larvae can avoid long-distance drift into unsuitable habitat and relocate their natal region from a greater distance.

The use of a time-compensated sun compass would be particularly valuable to species on isolated islands in steady ocean currents, where loss of sensory contact with the home reef is lethal and an innate swimming direction could easily evolve. We predict that fish tested at different locations with differently directed drift currents will show time-compensated, sun compass directed swimming in correspondingly different directions. We might even suggest that the combination of sun-compass-based directed swimming and natal reef recognition could be a significant factor in explaining the extraordinary biodiversity of coral reef fish species. Innate orientation supports homing, which can lead to genetic isolation and potential speciation as observed in migratory birds [41].

## Materials and Methods

### Ethics Statement

Fish collection permits were obtained from the Great Barrier Reef Marine Park Authority (G10/33239.1) and Queensland Department of Primary Industries and Fisheries (103256). The research was carried out in accordance with the Australian Code of Practice for the care and use of animals for scientific purposes. The protocol was approved by the Ethics Review Committee of James Cook University (Permit Number: A1614). All efforts were made to minimize suffering.

### Experimental Animals

The experiments were performed at the Field Station on One Tree Island (23.30′00 S, 152.05′00 E), a reef in the Capricorn-Bunker group of Islands in the southern Great Barrier Reef, Australia (Figure 1). In 2011, we collected newly settled *Ostorhinchus doederleini* on “patch-reefs” that were constructed from small coral rubble piles equivalent to natural substrates for settling *O. doederleini* [42]. The patch reefs (dimensions ∼1 m×1 m×0.5 m) were located on sand areas in one of the main entrances into the lagoon, 10 to 80 m from a main reef in 4–5 m water depth. Since settlement takes place at night [34] and we cleared the patch reefs early each day, the collected fish would only have been post-settlement for a few hours. They had pelagic (no-stripe) pigmentation. Using the otolith daily aging technique, we determined that the mean age of settlers that had arrived at the same time as the tested fish was 20.5 days, ranging from 16 to 35 days (n = 30). This overlaps with the youngest age of newly settled fish found at patch reefs in the lagoon [42].

In 2012, true pre-settlement *O. doederleini* were caught with channel nets that were set to fish on the flood tides only at night on 20^th^ and 21^st^ of January 2012; current speeds were 35 cm/sec or more. The nets (n = 2) were square mouthed (0.75×0.75 m), the mesh was 500 µm, organized as a box/pyramid with an efficiency of 1∶12 (mouth area: open sifting surface; full procedures see [43]). Pre-settlement *O. doederleini* were easily identified by morphology and a black dot on the caudal peduncle.

The collected fish measured 10–13 mm at the beginning of the experiments and 11–15 mm at the end of the experiments. The fish were kept in individual tanks made of plastic or glass. Each tank contained one piece of dead coral and fresh OTI Reef water, which was oxygenated with a bubble stone and partly changed once every 24 hours. The fish tanks were kept in a wet lab with an opening pointing towards N and translucent plastic “windows” on parts of the east and west sides allowing light to come in but through which no clear contours could be observed. The fish never had access to direct views of the sun from within their holding tanks but the non-clock-shifted fish will in some cases have been able to see parts of blue sky towards the North. Once per day, the fish were fed plankton caught during night-time plankton tows.

### Orientation Tests

Orientation tests with these ∼12 mm larvae were performed in a clear plastic circular bowl (17 cm [diameter] × 12 cm [depth]) allowing the fish a good view of the sky all the way down to the horizon. This bowl was placed on a leveled wooden platform on which a sundial of black lines was drawn in 22.5 degree segments relative to magnetic North. A finer angular resolution for recording fish position does not make sense given the size of the fish and the size of the bowl, but with 40 directions recorded, this resolution is more than sufficient to record the fish’s preferred direction. Each fish was tested separately. A given test was performed as follows: The fish was transferred from its home aquarium in the wet lab to the testing bowl on the beach with a small glass jar. It was carefully released into the middle of the bowl. After ca. 60 sec of acclimation, its geographic position relative to the centre of the bowl was recorded in 30 sec intervals for the next 20 min. The observer simply noted down, which of the black lines in the sun dial was closest to the head of the fish.

The fish spent the vast majority of their time hovering somewhere near the edge of the bowl. The fish position relative to the centre of the bowl was used as the directional measure rather than their heading, since they can move no further in any given direction once it reaches the edge of the bowl. Most of our fish clearly showed a preferred direction by either hovering fairly stationary or by slowly swimming back-and-fourth along the edge of the bowl around their preferred direction, or by moving away and quickly returning to the edge near the preferred direction. This behavior confirms that position in the bowl relative to the centre of the bowl is the most relevant orientation measure.

The observer sat on a stool next to the bowl. The observer systematically rotated position relative to the bowl (N, E, S, or W) between tests, so that the observer sat north of the bowl during 25% of the tests, south of the bowl in 25% of the tests, east of the bowl in 25% of the tests, and west of the bowl in 25% of the tests. The consistency of the fish’s orientation (Figure 2A–D and Figure S1 in File S1) shows that the position of the observer had no significant effect on the directional choices of the fish (see Figure S3 in File S1). If a strong observer bias would have existed, the distributions in Figure 2A–D should have been random or quadrimodal since the observer position was balanced between N, S, E, and W. In sum, observer bias could never have *improved* the clarity of the results. In contrast, any slight observer bias would have added a symmetrical distribution component to the results and therefore *reduced* the clarity of the observed directional responses.

At the end of the test, the fish was transferred back to its holding tank. Between tests, the water in the test bowl was replaced by fresh OTI reef sea water.

Individual fish were tested at least three times (3–5 times) in each condition (for details see supplementary information, Tables S1–S4 in File S1). It is known from the vast literature on orientation tests with migratory birds that repeated tests are needed to determine the intended mean orientation direction of an individual with reasonable accuracy (e.g. [44], [45], [46], [47], [48]). By using average directions based on repeated tests of the same individuals, the number of experimental animals can also be significantly reduced, which is an important ethical consideration in modern biology.

### Clock-shifting

Five newly settled fish in 2011 and all 7 pre-settlement fish in 2012 were clock-shifted after they had been tested and shown clear orientation in three control tests under non-clock-shifted conditions. The fish were clock-shifted 6 h backwards by placing them in a windowless room, where the light (5–7 lamps fitted with a mix of incandescent and energy-saving bulbs and connected to an automatic timer) went on at 23∶15 and off at 13∶15. Water changes and feeding (plankton, see above) took place in the morning between 7–10. While the five 2011 fish were being clock-shifted and then tested outdoors under natural sunny skies, we continued to keep and test the other nine fish from the same cohort under non-clock-shifted conditions. Thereby, we ensured that there were no time-in-captivity effects on the orientation direction of the larvae. Also, we took care to perform the first tests with the clock-shifted fish quite late in the morning (details in Table S3 in File S1), so that even if the fish’s clock was not yet shifted the full 6 hours (we expect the clock-shift to have been complete after 6 days, but just in case), the predicted change in orientation would still be close to 180 degrees (compare sun azimuth position late in the morning, e.g. between 09∶00 and 11∶00 with the sun azimuth 5 hours later in Figure 2E). Finally, we took care to test the clock-shifted fish outdoors under the natural sunny sky at the exact same location where we performed the non-clock-shifted tests. Since all individual fish clearly shifted their orientation by ca. 180° in response to the clock-shift (Figure 3), the orientation of the fish were not affected by local landmarks visible through the sides of the bowl.

### Evaluation of Results and Circular Statistics

The evaluation of the orientation data was done using the standard methods applied in animal orientation studies [49]. The circular statistics program Oriana was used to calculate the mean and concentration of the 40 observed directions. All orientation directions recorded were corrected for the declination at One Tree Island (by turning the results 10 degrees counter-clockwise; e.g. an orientation towards 167 degrees relative to magnetic North is equivalent to 157 degrees relative to geographic North at One Tree Island) so that North in all figures and calculations refers to geographical North, not magnetic North, as geographical North is the relevant reference for a sun compass.

Since we cannot completely exclude that each individual observation may be somewhat dependent on the previous location of the fish, for the circular diagrams, we only included the results of single tests where the orientation of the fish was very clearly directed (very clearly directed = tests where the directionality was significant at the p<0.001 level according to the Rayleigh test; [49]). This criterion was reached in 114 out of 132 individual tests (86%, see Table S1 and Table S3 in File S1). Of the 18 tests that did not reach this very conservative significance level, 10 were performed between 11∶15 and 12∶45, when the sun is so high in the sky and the sun azimuth changes so fast that it is unlikely that a sun compass would work, see Figure 2E). Thus, only 8 individual tests (6% of the 132 tests), done when the sun azimuth was likely to have provided useful information, resulted in disoriented behavior.

To give the reader the clearest impression of the variability in the test results, in the supplementary materials (File S1), we depict the data in two different ways. In Figure S1A, S1C, S1E and S1G in File S1, we include the results of all individual tests. In this kind of illustration, several separate tests of the same fish are included and thus the stated “n” is pseudo-replicated. However, it is known from orientation experiments with birds that the inter- and intra-individual variation in orientation are similar [45]. The fact that the 95% confidence intervals in our experiments remained almost identical regardless whether all tests or individual means are depicted (compare Figure S1A with S1B, S1C with S1D, S1E with S1F, and S1G with S1H, all in File S1) shows that intra- and inter-individual variation are also similar for our fish. Even though some readers may find this additional graph superfluous, we find it important to also present the results of all individual tests in a circular diagram, since it gives the reader a good impression of the spread in orientation observed between tests.

The second order mean directions of 3–5 tests per individual (Figure 2A–D, and Fig. S1B, S1D, S1F, and S1H in File S1) were calculated by vector addition of unit vectors in each of the mean directions from the individual tests (see Table S4 in File S1). This is the standard procedure used in the literature on migratory birds tested in orientation cages for the last four decades (e.g. [44], [46], [47], [48], [49]).

Time-of-day effects (Figure 2F) on the directedness of the individual tests were evaluated post hoc resulting in slightly different sample sizes. As a measure for directedness we use the length of the mean vector, “r”, for each individual test. The r-value is a measure of how consistently a given fish oriented in its chosen mean direction during a single 20-minute test session. This analysis is based on the logic that if a fish has access to good orientation cues, it should orient more consistently in whatever direction it chooses than if a fish has access to poor orientation relevant information, e.g. because the azimuth angle is difficult to determine due to the sun being very close to the zenith. To avoid any concern about pseudoreplication in this post hoc analysis, the average r-value of the two tests was entered into the calculations if a given fish had been tested twice during the same time-period. The data leading to Figure 2F are provided in supplementary materials (Table S2 in File S1).

### Genetic Analysis

Tissue of test animals was stored in 99% ethanol until DNA extraction. DNA was isolated from the samples using Chelex chelating resin (BioRad Chelex 100 Resin). We used five previously described polymorphic DNA microsatellite markers: Ad65.2, Ad67 Ad70 Ad86.2, Ad94 Table 1 [50]. PCR was carried out using approximately 100 ng of template DNA and the following cycle treatment; initial step of 5 min at 94°C, followed by 35 cycles of 30 s at 94°C, 30 s at 44–46°C, and 1 min at 72°C, with a final extension step of 5 min at 72°C. Total reaction volume was 10 µL and contained 2.5 µL 10 X RED Taq Polymerase Buffer (Sigma, 10 mM Tris-HCl, pH 8.3, 50 mM KCl, 1.1 mM MgCl2 and 0.01% gelatin), 100 µM of each dNTP (Promega), 0.5 µM of both forward and reverse primer, and 0.5 U Taq polymerase (Sigma) 0.25 U RED Taq DNA Polymerase (Sigma). PCR fragments were separated and scored on a Beckman-Coulter CEQTM 2000XL DNA analysis system.

Sun compass tested juveniles were statistically assigned to adult populations of adjacent reefs (OTI, Lamont, heron, Fitzroy reefs) using Geneclass2 [51].

## Supporting Information

File S1 **Supporting Figures S1, S2, S3, and Supporting Tables S1, S2, S3, and S4.**
(PDF)
