# Supplementary material for: Sun Compass Orientation Helps Coral Reef Fish Larvae Return to Their Natal Reef
Source: PLoS One. 2013 Jun 26;8(6):e66039. doi: 10.1371/journal.pone.0066039 (PMC3694079; doi:10.1371/journal.pone.0066039)
Supplement: File S1 — Supporting Figures S1, S2, S3, and Supporting Tables S1, S2, S3, and S4. (PDF) [file pone.0066039.s001.pdf]

Supplementary Material

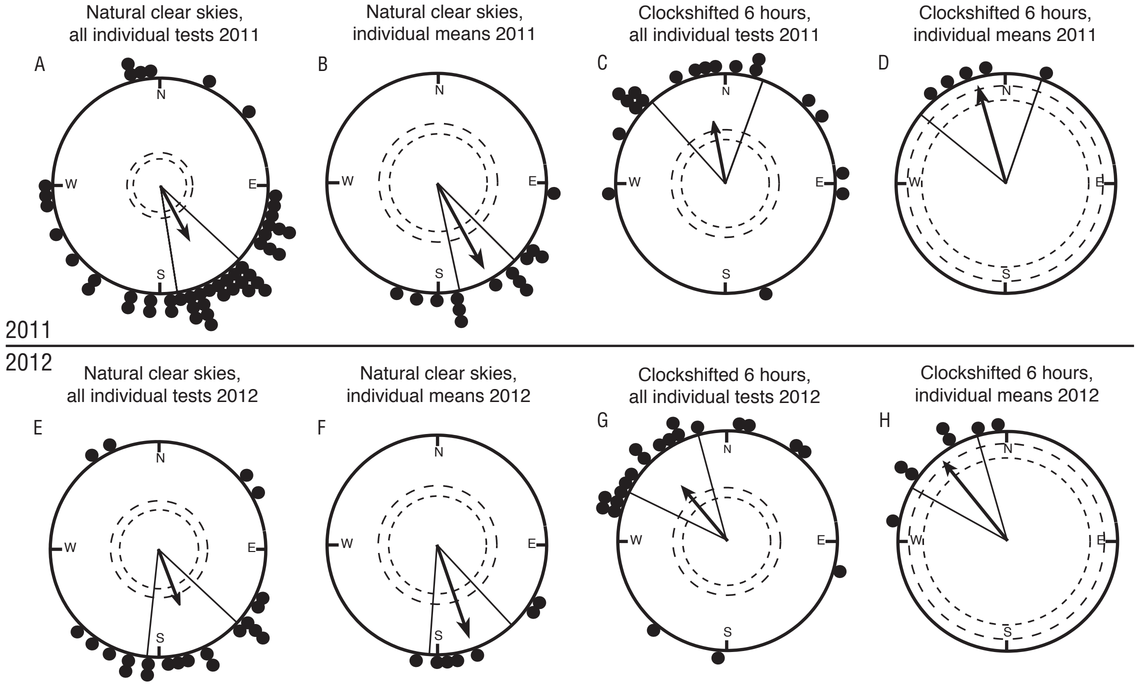

Figure S1. Inter- and intra-individual variation in orientation is similar in our settling stage *Ostorhinchus doederleini*. A: Forty-nine single tests under natural sunny skies with 14 just-settled *O. doederleini* tested in 2011 showed a clear orientation towards SSE, both when all the individual tests were considered (A: mean direction: 162°, r=0.56, n=49, p<0.001) and when only the second order means of each of the tested individuals were considered (B: mean direction: 162°, r=0.88, n=14, p<0.001). When five fish were clock-shifted 6 hours backwards, they turned their orientation by ca. 180° (C: all individual tests, mean direction: 359°, r=0.58, n=19, p<0.001; D: individual second order means, mean direction: 354°, r=0.94, n=5, p<0.01). Twenty-two single tests under natural sunny skies with 7 pre-settlement *O. doederleini* tested in 2012 also showed a clear orientation towards SSE, both when all the individual tests were considered (E: mean direction: 169°, r=0.57, n=22, p<0.001) and when only the second order means of each of the tested individuals were considered (F: mean direction: 171°, r=0.91, n=7, p<0.001). When all 7 fish were clock-shifted 6 hours backwards, they turned their orientation by ca. 180° (G: all individual tests, mean direction: 330°, r=0.62, n=21, p<0.001; H: individual second order means, mean direction: 331°, r=0.92, n=7, p<0.001). Notice that the 95% confidence intervals (lines flanking the group mean vector) in our experiments remained almost identical regardless whether all tests or individual means are depicted (compare Fig. S1A with S1B, S1C with S1D, S1E with S1F, and S1G with S1H). This is only to be expected if the intra- and inter-individual variation in orientation is very similar for our fish. Each dot at the circle periphery indicates the mean orientations observed in each of the individual tests done under the given condition (A, C, E, G) or the mean orientation chosen by each individual fish based on the second order average of all tests of the given fish in the given condition (B, D, F, H). Arrows indicate the group mean vectors. Inner and outer dashed circles indicate the radius of the group mean vector needed for significance according to the Rayleigh Test (p<0.05 and p<0.01, respectively).

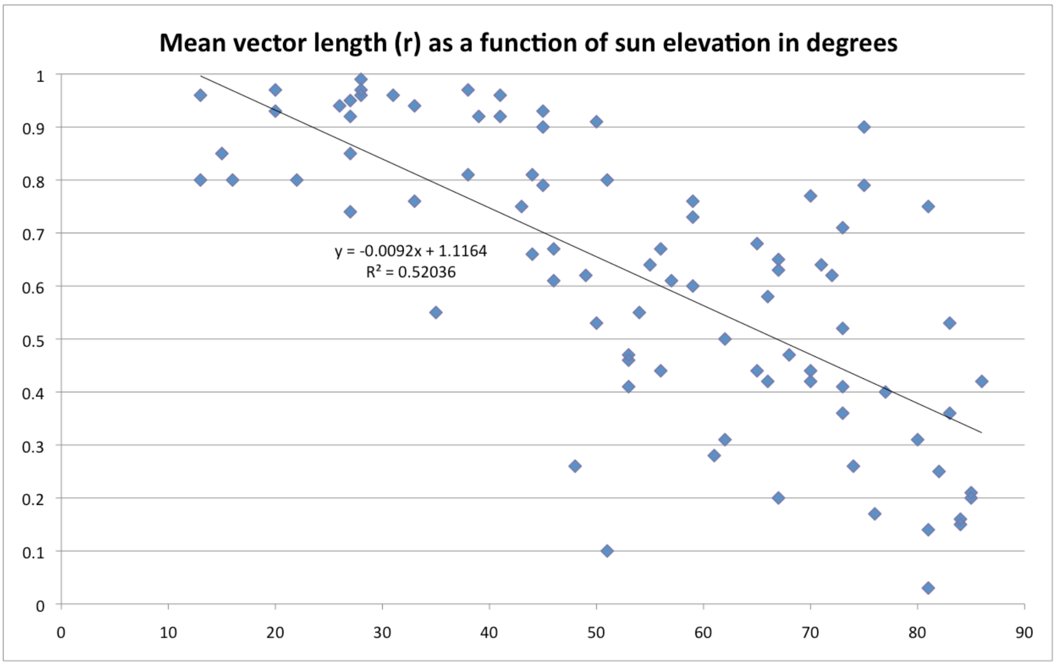

Figure S2. The lower the sun elevation, the better the fish were able to keep their chosen heading. The y-axis indicates the r-value for each individual test (the length of the mean vector of the 40 recorded single direction in a given single test). The x-axis indicates the sun elevation in degrees. There is a highly significant negative correlation between the r-value and the sun elevation, which means that the higher the sun elevation is, the poorer the fish are able to keep a chosen direction.

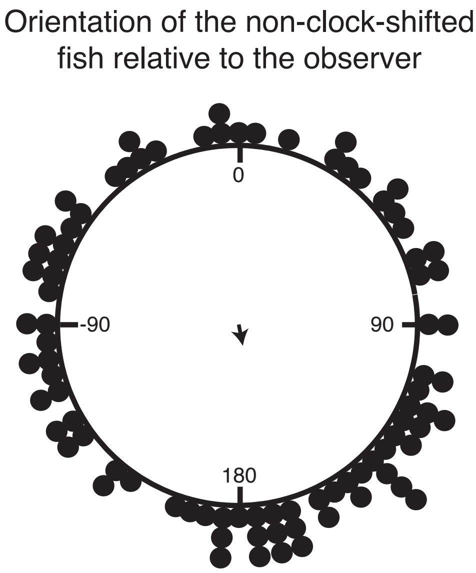

Figure S3. Evidence for no significant observer bias. The observer's position is defined as 0 degrees and the orientation of each individual fish in the non-clock-shifted condition is indicated by the dots at the circle periphery (2011 and 2012 fish combined). Thus, a point in 0 degrees would mean that the fish oriented towards the observer and a point in 180 degrees would mean that the fish oriented away from the observer. The distribution is random (mean direction 169°, r=0.12, n=87, n.s.). For description of the circular diagram, see legend to Figure S1.

Table S1. Orientation results for non-clock-shifted larvae. “Time tested” indicates the start of the given 20 minute test. “r” is the length of the mean vector calculated from the 40 individual directions recorded during the individual test. “p” is the p-value resulting from a Rayleigh Test. However, since each individually recorded direction is unlikely to be completely independent, to be conservative, we demanded a p<0.001 for inclusion of the direction in the circular diagrams (Figure 1A-D and Figure S1).

Table S1.									
Fish ID	Date tested (D.M.Y)	Time tested	Orientation relative to magnetic North in degrees	Orientation relative to geographic North in degrees	r	"p"	Sun height above horizon in degrees	Sun azimuth in degrees	Comment (NS = considered non-significant)
HM4	20.1.2011	10:17	119	109	0.63	0.001	67	86	
HM5	20.1.2011	12:00	187	177	0.42	0.001	86	331	
HM2	20.1.2011	13:07	169	159	0.79	0.001	75	279	
HM1	20.1.2011	15:34	154	144	0.96	0.001	41	263	
HM3	20.1.2011	16:34	180	170	0.99	0.001	28	259	
HM4	21.1.2011	08:51	132	122	0.67	0.001	46	95	
HM2	21.1.2011	09:22	3	353	0.47	0.001	53	93	
HM5	21.1.2011	12:36	256	246	0.75	0.001	81	293	
HM1	21.1.2011	13:10	201	191	0.26	0.07	74	279	NS
HM3	21.1.2011	15:03	29	19	0.26	0.06	48	266	NS
HM3	22.1.2011	8:44	107	97	0.81	0.001	44	95	
HM1	22.1.2011	9:17	326	316	0.10	0.68	51	93	NS
HM5	22.1.2011	9:48	128	118	0.60	0.001	59	90	
HM2	22.1.2011	10:52	149	139	0.71	0.001	73	81	
HM4	22.1.2011	13:48	270	260	0.68	0.001	65	274	
HM3	22.1.2011	15:49	273	263	0.81	0.001	38	263	
HM1	22.1.2011	16:38	147	137	0.85	0.001	27	259	
HM1	23.1.2011	8:42	147	137	0.75	0.001	43	95	
HM10	24.1.2011	10:43	129	119	0.64	0.001	71	81	
HM11	24.1.2011	11:11	121	111	0.40	0.001	77	74	
HM12	24.1.2011	11:39	201	191	0.36	0.01	83	55	NS
HM13	24.1.2011	14:04	268	258	0.31	0.02	62	273	NS
HM9	24.1.2011	14:43	203	193	0.46	0.001	53	269	
HM12	25.1.2011	14:39	117	107	0.55	0.001	54	270	
HM13	25.1.2011	15:11	281	271	0.61	0.001	46	267	
HM9	25.1.2011	15:50	355	345	0.97	0.001	38	263	
HM10	25.1.2011	16:17	174	164	0.96	0.001	31	261	
HM11	25.1.2011	16:43	357	347	0.94	0.001	26	259	
HM10	26.1.2011	10:24	37	27	0.42	0.001	66	84	
HM12	26.1.2011	10:52	61	51	0.36	0.001	73	78	
HM13	26.1.2011	11:33	338	328	0.14	0.44	81	59	NS
HM11	26.1.2011	12:00	258	248	0.21	0.18	85	12	NS
HM9	26.1.2011	12:26	224	214	0.53	0.001	83	311	
HM11	26.1.2011	15:33	359	349	0.92	0.001	41	265	
HM13	26.1.2011	16:02	160/340	-	0.55	0.001	35	263	bimodal
HM9	26.1.2011	16:36	186	176	0.92	0.001	27	260	
HM12	26.1.2011	17:30	163	153	0.85	0.001	15	256	
HM9	27.1.2011	11:08	317	307	0.17	0.3	76	72	NS
HM12	27.1.2011	11:35	188	178	0.25	0.09	82	56	NS
HM10	27.1.2011	12:02	165	155	0.20	0.19	85	6	NS
HM10	27.1.2011	16:34	161	151	0.97	0.001	28	261	

HM13	28.1.2011	16:30	171	161	0.96	0.001	28	261	
HM20	28.1.2011	16:58	207	197	0.80	0.001	22	259	
HM19	28.1.2011	17:26	171	161	0.80	0.001	16	257	
HM19	29.1.2011	11:31	193	183	0.31	0.02	80	57	NS
HM20	29.1.2011	11:55	97	87	0.15	0.42	84	23	NS
HM13	29.1.2011	12:18	307	297	0.16	0.37	84	330	NS
HM19	29.1.2011	13:16	224	214	0.52	0.001	73	285	
HM20	29.1.2011	13:45	241	231	0.58	0.001	66	278	
HM9	29.1.2011	14:55	194	184	0.91	0.001	50	270	
HM11	29.1.2011	15:18	174	164	0.93	0.001	45	268	
HM12	30.1.2011	9:12	160	150	0.62	0.001	49	90	
HM19	30.1.2011	9:45	147	137	0.61	0.001	57	87	
HM13	30.1.2011	10:33	124	114	0.47	0.001	68	80	
HM22	31.1.2011	11:56	18	8	0.03	0.96	81	50	NS
HM21	31.1.2011	13:11	169	159	0.41	0.001	73	288	
HM20	31.1.2011	13:40	121	111	0.20	0.22	67	281	NS
HM22	31.1.2011	14:28	177	167	0.67	0.001	56	273	
HM12	31.1.2011	14:51	147	137	0.80	0.001	51	271	
HM20	31.1.2011	15:22	153	143	0.66	0.001	44	268	
HM11	1.2.2011	8:55	148	138	0.90	0.001	45	91	
HM22	1.2.2011	9:54	152	142	0.73	0.001	59	85	
HM21	1.2.2011	14:02	109	99	0.50	0.001	62	278	
HM22	1.2.2011	14:32	121	111	0.64	0.001	55	273	
HM21	1.2.2011	15:15	195	185	0.79	0.001	45	269	
OTI20	20.1.2012	10:37	193	183	0.77	0.001	70	85	
OTI21	20.1.2012	11:01	138	128	0.90	0.001	75	80	
OTI22	20.1.2012	13:17	203	193	0.62	0.001	72	277	
OTI23	20.1.2012	13:42	333	323	0.65	0.001	67	273	
OTI24	20.1.2012	14:05	180	170	0.28	0.04	61	271	NS
OTI20	20.1.2012	14:30	71	61	0.44	0.001	56	268	
OTI21	20.1.2012	14:53	180	170	0.53	0.001	50	266	
OTI22	20.1.2012	15:18	194	184	0.79	0.001	45	264	
OTI23	20.1.2012	15:42	213	203	0.92	0.001	39	262	
OTI24	20.1.2012	16:08	142	132	0.94	0.001	33	261	
OTI20	20.1.2012	16:08	129	119	0.76	0.001	33	261	
OTI21	20.1.2012	16:37	237	227	0.95	0.001	27	258	
OTI22	20.1.2012	16:37	60	50	0.74	0.001	27	258	
OTI24	20.1.2012	17:09	126	116	0.93	0.001	20	256	
OTI23	20.1.2012	17:09	173	163	0.97	0.001	20	256	
OTI23	20.1.2012	17:38	147	137	0.80	0.001	13	254	
OTI24	20.1.2012	17:38	138	128	0.96	0.001	13	254	
OTI26	21.1.2012	9:23	166	156	0.47	0.001	53	93	
OTI25	21.1.2012	9:23	164	154	0.41	0.001	53	93	
OTI26	21.1.2012	9:50	206	196	0.76	0.001	59	90	
OTI25	21.1.2012	10:14	184	174	0.44	0.001	65	88	
OTI26	21.1.2012	10:38	225	215	0.42	0.001	70	84	
OTI25	21.1.2012	10:38	346	336	0.44	0.001	70	84	

Table S2. Mean vector lengths (r) in individual orientation tests performed in different time of day intervals for the non-clock-shifted larvae tested in 2011. Data originates from Table S1 (column 6). The mean time for any given 20 min test (i.e. “Time tested” + 10 min) decided in which time interval the test was considered. The third column in this table gives the mean r-value for each individual fish that was tested in a given time interval, and these values were used in the calculations of the means and standard deviations as indicated on Figure 1F and for the subsequent ANOVA.

Table S2

Fish ID	r-value in single tests	Mean r-value used for Figure 1F
8:00-9:45		
HM1	0.10	0.43
	0.75	
HM2	0.47	0.47
HM3	0.81	0.81
HM4	0.67	0.67
HM11	0.90	0.90
HM12	0.62	0.62
9:45-11:15		
HM2	0.71	0.71
HM4	0.63	0.63
HM5	0.60	0.60
HM10	0.64	0.53
	0.42	
HM12	0.36	0.36
HM13	0.47	0.47
HM19	0.61	0.61
HM22	0.73	0.73
11:15-12:45		
HM5	0.42	0.42
HM9	0.53	0.35
	0.17	
HM10	0.20	0.20
HM11	0.40	0.31
	0.21	
HM12	0.36	0.30
	0.25	
HM13	0.14	0.15
	0.16	
HM19	0.31	0.31
HM20	0.15	0.15
HM22	0.03	0.03
**Table S2 (continued)**		
12:45-14:15		
HM1	0.26	0.26
HM2	0.79	0.79
HM4	0.68	0.68
HM5	0.75	0.75
HM13	0.31	0.31
HM19	0.52	0.52
HM20	0.58	0.39
	0.2	
HM21	0.41	0.46
	0.5	
14:15-15:45		
HM3	0.26	0.26
HM9	0.46	0.68
	0.91	
HM11	0.92	0.93
	0.93	
HM12	0.55	0.67
	0.80	
HM13	0.61	0.61
HM20	0.66	0.66
HM21	0.79	0.79
HM22	0.67	0.66
	0.64	
after 15:45		
HM1	0.96	0.90
	0.85	
HM3	0.99	0.90
	0.81	
HM9	0.97	0.94
	0.92	
HM10	0.96	0.97
	0.97	
HM11	0.94	0.94
HM12	0.85	0.85
HM13	0.55	0.75
	0.96	
HM19	0.80	0.80
HM20	0.80	0.80

Table S3. Orientation results for clock-shifted larvae. "Time tested" indicates the start of the given 20 minute test. "r" is the length of the mean vector calculated from the 40 individual directions recorded during the individual test. "p" is the p-value resulting from a Rayleigh Test. However, since each individually recorded direction is unlikely to be completely independent, to be conservative, we demanded a p<0.001 for inclusion of the direction in the circular diagrams (Figure 1A-D and Figure S1).

Table S3.

Fish ID	Date tested	Time tested	Orientation relative to magnetic North in degrees	Orientation relative to geographic North in degrees	r	"p"	Sun height above horizon in degrees	Sun azimuth in degrees	Sun azimuth 6 hours later in degrees	Predicted clockwise shift in degrees	Comment
HM1 (cs)	29.1.2011	8:19	5	355	0.50	0.001	37	95	276	179	
HM3 (cs)	29.1.2011	8:48	318	308	0.56	0.001	44	93	270	183	
HM4 (cs)	29.1.2011	9:17	168	158	0.43	0.001	51	90	268	182	
HM5 (cs)	29.1.2011	9:50	106	96	0.57	0.001	58	87	265	182	
HM4 (cs)	29.1.2011	10:26	26	16	0.91	0.001	66	81	262	179	
HM5 (cs)	30.1.2011	8:24	23	13	0.75	0.001	38	95	273	182	
HM4 (cs)	30.1.2011	8:51	93	83	0.11	0.61	45	92	270	182	NS
HM3 (cs)	30.1.2011	11:02	17	7	0.55	0.001	74	72	259	173	
HM1 (cs)	31.1.2011	9:39	64	54	0.54	0.001	55	87	268	179	
HM4 (cs)	31.1.2011	10:03	323	313	0.44	0.001	61	84	264	180	
HM5 (cs)	31.1.2011	10:28	325	315	0.84	0.001	67	80	262	178	
HM3 (cs)	31.1.2011	10:52	93	83	0.43	0.001	72	74	260	174	
HM4 (cs)	1.2.2011	6:45	306	296	0.66	0.001	16	102	302	160	
HM1 (cs)	1.2.2011	7:09	1	351	0.90	0.001	21	100	290	170	
HM5 (cs)	1.2.2011	7:34	277	267	0.66	0.001	27	98	283	175	
HM3 (cs)	1.2.2011	8:04	319	309	0.60	0.001	34	96	277	179	
HM10 (cs)	1.2.2011	8:30	353	343	0.83	0.001	40	94	274	180	
HM10 (cs)	1.2.2011	9:19	347	337	0.62	0.001	51	89	269	180	
HM10 (cs)	1.2.2011	10:17	318	308	0.50	0.001	64	81	263	178	
HM1 (cs)	1.2.2011	10:43	55	45	0.72	0.001	70	76	261	175	
OTI20 (cs)	25.1.2012	10:25	324	314	0.56	0.001	67	84	261	183	
OTI21 (cs)	25.1.2012	10:25	299	289	0.57	0.001	67	84	261	183	
OTI22 (cs)	25.1.2012	10:50	195	185	0.46	0.001	72	79	259	180	
OTI23 (cs)	25.1.2012	10:50	325	315	0.57	0.001	72	79	259	180	
OTI24 (cs)	26.1.2012	7:16	303	293	0.90	0.001	24	101	282	179	
OTI20 (cs)	26.1.2012	7:16	49	39	0.94	0.001	24	101	282	179	
OTI21 (cs)	26.1.2012	7:43	298	288	0.95	0.001	30	99	277	182	
OTI22 (cs)	26.1.2012	7:43	344	334	0.71	0.001	30	99	277	182	
OTI23 (cs)	26.1.2012	8:11	309	299	0.95	0.001	36	97	273	184	
OTI24 (cs)	26.1.2012	8:11	17	7	0.91	0.001	36	97	273	184	
OTI20 (cs)	26.1.2012	8:37	239	229	0.19	0.24	42	95	270	185	NS
OTI21 (cs)	26.1.2012	8:37	353	343	0.65	0.001	42	95	270	185	
OTI23 (cs)	26.1.2012	9:04	232	222	0.79	0.001	48	93	268	185	
OTI22 (cs)	26.1.2012	9:04	335	325	0.96	0.001	48	93	268	185	
OTI20 (cs)	26.1.2012	9:35	297	287	0.88	0.001	55	90	265	185	
OTI24 (cs)	26.1.2012	9:35	44	34	0.91	0.001	55	90	265	185	
OTI25 (cs)	26.1.2012	10:00	115	105	0.54	0.001	61	87	263	184	
OTI26 (cs)	26.1.2012	10:00	297	287	0.78	0.001	61	87	263	184	
OTI26 (cs)	26.1.2012	10:26	22	12	0.67	0.001	67	83	261	182	
OTI25 (cs)	26.1.2012	10:26	342	332	0.56	0.001	67	83	261	182	
OTI26 (cs)	27.1.2012	8:05	344	334	0.89	0.001	34	97	274	183	

OTI25 (cs)	27.1.2012	8:05	314	304	0.78	0.001	34	97	274	183	
OTI25 (cs)	27.1.2012	10:05	354	344	0.71	0.001	62	86	263	183	
OTI26 (cs)	27.1.2012	10:05	339	329	0.77	0.001	62	86	263	183	
										Mean 180, r=1.00(!), P<0.001	

Table S4: Summary table for calculations of individual mean orientation before and after clock-shift. The individual mean directions were calculated by vector addition of unit vectors in each of the mean directions shown in the individual tests of the given individual (Batchelet 1981).

Table S4.						
Fish ID	Means orientation of individual non-clock-shifted tests	Mean orientation of the individual in the non-clock-shifted tests	Clock-shifted from date (D.M.Y)	Mean orientation of individual clock-shifted tests	Mean orientation of the individual in the clock-shifted tests	Clockwise deviation in the clock-shifted condition compared to the orientation of the same individual in the non-clock-shifted condition
HM1	144, 137, 137	139	23.1.2011	355, 54, 351, 45	21	200
HM2	159, 353, 139	128	22.1.2011	Died during clock-shifting	-	-
HM3	170, 97, 263	172	22.1.2011	308, 7, 83, 309	349	177
HM4	109, 122, 260	142	22.1.2011	158, 16, 313, 296	320	178
HM5	177, 246, 118	179	22.1.2011	96, 13, 315, 267	342	163
HM9	193, 345, 214, 176, 184	200	-	Not clock-shifted	-	-
HM10	119, 164, 27, 151	124	27.1.2011	343, 337, 308	329	205
HM11	111, 347, 349, 164, 138	94	-	Not clock-shifted	-	-
HM12	107, 51, 153, 150, 137	123	-	Not clock-shifted	-	-
HM13	271, 161, 114	170	-	Not clock-shifted	-	-
HM19	161, 214, 137	170	-	Not clock-shifted	-	-
HM20	197, 231, 143	191	-	Not clock-shifted	-	-
HM21	159, 99, 185	149	-	Not clock-shifted	-	-
HM22	167, 142, 111	140	-	Not clock-shifted	-	-
OTI20	183, 61, 119	120	21.1.2012	314, 39, 287	329	209
OTI21	128, 170, 227	174	21.1.2012	289, 288, 343	306	132
OTI22	193, 184, 50	160	21.1.2012	185, 334, 325	303	143
OTI23	323, 203, 163, 137	181	21.1.2012	315, 299, 222	281	100
OTI24	132, 116, 128	125	21.1.2012	293, 7, 34	354	229
OTI25	154, 174, 336	172	21.1.2012	105, 332, 304, 344	344	172
OTI26	156, 196, 215	189	21.1.2012	287, 12, 334, 329	331	142

Batchelet (1981). Circular Statistics in Biology. London: Academic Press.
